# Targeting Viral cccDNA for Cure of Chronic Hepatitis B

**DOI:** 10.1007/s11901-020-00534-w

**Published:** 2020-07-10

**Authors:** Gaëtan Ligat, Kaku Goto, Eloi Verrier, Thomas F. Baumert

**Affiliations:** 1 Université de Strasbourg, 67000 Strasbourg, France; 2 Institut de Recherche sur les Maladies Virales et Hépatiques, Inserm U1110, 3 Rue Koeberlé, 67000 Strasbourg, France; 3 Institut Hospitalo-Universitaire, Pôle Hépato-digestif, Nouvel Hôpital Civil, 67000 Strasbourg, France

**Keywords:** Antivirals, Drug discovery and development, HBV cure, Therapeutics

## Abstract

**Purpose of Review:**

Chronic hepatitis B (CHB), caused by hepatitis B virus (HBV), is a major cause of advanced liver disease and hepatocellular carcinoma (HCC) worldwide. HBV replication is characterized by the synthesis of covalently closed circular (ccc) DNA which is not targeted by antiviral nucleos(t)ide analogues (NUCs) the key modality of standard of care. While HBV replication is successfully suppressed in treated patients, they remain at risk for developing HCC. While functional cure, characterized by loss of HBsAg, is the first goal of novel antiviral therapies, curative treatments eliminating cccDNA remain the ultimate goal. This review summarizes recent advances in the discovery and development of novel therapeutic strategies and their impact on cccDNA biology.

**Recent Findings:**

Within the last decade, substantial progress has been made in the understanding of cccDNA biology including the discovery of host dependency factors, epigenetic regulation of cccDNA transcription and immune-mediated degradation. Several approaches targeting cccDNA either in a direct or indirect manner are currently at the stage of discovery, preclinical or early clinical development. Examples include genome-editing approaches, strategies targeting host dependency factors or epigenetic gene regulation, nucleocapsid modulators and immune-mediated degradation.

**Summary:**

While direct-targeting cccDNA strategies are still largely at the preclinical stage of development, capsid assembly modulators and immune-based approaches have reached the clinical phase. Clinical trials are ongoing to assess their efficacy and safety in patients including their impact on viral cccDNA. Combination therapies provide additional opportunities to overcome current limitations of individual approaches.

## Introduction

Chronic hepatitis B (CHB), caused by hepatitis B virus (HBV), is a worldwide leading cause of liver fibrosis, cirrhosis and hepatocellular carcinoma (HCC) [1, 2]. HCC is currently the second cause of death from cancer, and more than 50% of HCC cases are related to HBV infection in the most affected geographic areas [2, 3*]. Despite the availability of an effective prophylactic vaccine, HBV remains a worldwide major public health concern with an estimated prevalence of 250–300 million infected people globally [4**].

Current treatments against CHB include nucleos(t)ide analogues (NUCs) (such as lamivudine or entecavir) and pegylated interferon-*α* (PEG-IFN-*α*) [5, 6]. NUCs have been proven to effectively control HBV infection by suppression of viral replication and thus improve quality of life and survival in patients; however, they are not curative. Furthermore, NUCs are generally long-term or lifelong treatments. PEG-IFN-*α*-based therapies can result in viral cure in a small subset of patients; however, these therapies are limited by a low response rate and significant side effects, which preclude a widespread application of this strategy [7]. Sustained viral replication and liver injury are recognized as key risk factors for HBV-related HCC, which are reduced by antiviral therapy [5, 6]. Nevertheless, successful viral control in treated patients reduces but does not eliminate the risk of HCC, whose annual incidences range from 0.9 to 5.4% under treatment with NUCs in the presence of cirrhosis [8]. Most approaches in clinical development aim for functional cure characterized by sustained loss of hepatitis B surface antigen with or without hepatitis B surface antibody seroconversion, which is associated with improved clinical outcomes [9, 10, 13]. Since persistent cccDNA acts as a reservoir for viral relapse [11**], eradication of HBV DNA including intrahepatic covalently closed circular DNA (cccDNA), i.e. complete sterilizing cure, is the ultimate goal [10, 12, 13]. However, sterilizing cure is much more difficult to achieve [10, 12, 13].

HBV infection into human hepatocytes is thought to be initiated by binding to heparan sulphate proteoglycans including glypican 5 (GPC5) [14**] with subsequent viral cell entry mediated by the sodium taurocholate cotransporting polypeptide (NTCP) with EGF receptor (EGFR) as a facilitator [15**, 16**, 17**]. The genome of HBV is a 3.2 kb relaxed circular (rc) DNA whose one of the two strands is covalently linked to the viral polymerase. Upon infection, the viral genome is translocated and released into the host cell nucleus where the rcDNA is converted into an episomal cccDNA. cccDNA is the transcriptional template for all viral gene products, including the pregenomic RNA (pgRNA). The pgRNA is selectively packaged into a capsid and then reverse transcribed into rcDNA. Mature nucleocapsid can be used for cccDNA amplification or be enveloped to release a generation of new virions [18*].

In the nucleus of infected hepatocytes, cccDNA persists as a stable minichromosome [19, 20] associated with histone and non-histone proteins [21]. Gene expression is regulated by cellular factors including transcription factors and chromatin-modifying enzymes [22]. Viral proteins HBc (core) and HBx support the structural and functional features of cccDNA [23]. It is noteworthy that a few cccDNA copies per liver cell are sufficient to reactivate viral multiplication after therapy withdrawal or loss of immunological control. Therefore, maintenance of cccDNA with ineffective immune responses leads to HBV chronicity [23].

Thus, the ideal therapeutic strategy for curative approaches includes reduction or elimination of the whole cccDNA pool. Addressing this issue, we review here how novel therapeutic strategies affect cccDNA in a direct or indirect manner including genome editing, epigenetics and gene regulation, host-targeting approaches, nucleocapsid assembly and immunity.

## Direct cccDNA Degradation

**Fig. 1:**
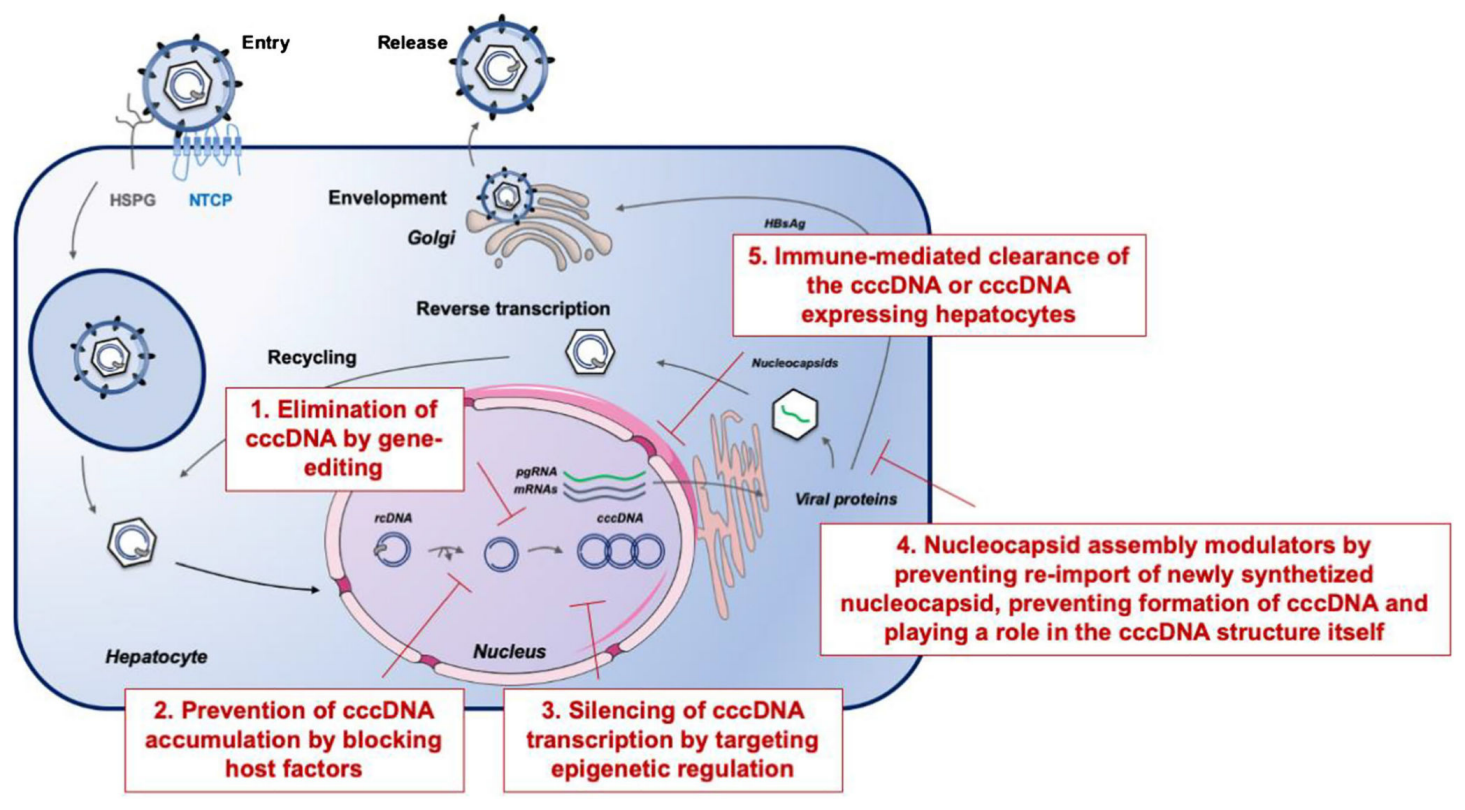
Therapeutic strategies and their potential impact on viral cccDNA within the HBV life cycle. Upon infection, the viral genome is translocated and released into the nucleus where the rcDNA is converted into an episomal covalently closed circular DNA (cccDNA). CHB is linked to the persistence of the cccDNA, and a few cccDNA copies per liver cell can reactivate full virus production after therapy withdrawal. Chronic hepatitis B cure is believed to require cccDNA elimination or functional knockout of cccDNA by silencing of cccDNA activity. Examples for strategies aiming for HBV cure include (1) elimination of cccDNA by gene editing, (2) prevention of cccDNA accumulation by blocking host factors involved in cccDNA formation, (3) silencing of cccDNA transcription by targeting epigenetic regulation, (4) nucleocapsid assembly modulators by preventing reimport of newly synthetized nucleocapsid and thereby prevent amplification of the cccDNA pool, preventing formation of cccDNA and maybe playing a role in the cccDNA structure itself and (5) immune-mediated clearance of the cccDNA or cccDNA expressing hepatocytes

The most direct anti-cccDNA strategy is its specific degradation, whose long-standing difficulty was recently overcome by new genome-editing technologies [24] (Fig. 1). The major editing systems, including the zinc finger nuclease (ZFN) [25], transcription activator-like effector nucleases (TALENs) [26] and the clustered regularly interspaced short palindromic repeats/CRISPR-associated (CRISPR/Cas) system [27], have been used to disrupt HBV cccDNA. All these editing systems create a DNA double-strand break in a specific target site and repair the cleavage sites by altering DNA sequence. Particularly, the CRISPR/Cas9 system was the most successful [28], arousing widespread interest according to its simplicity and flexibility [29]. CRISPR/Cas9-based anti-HBV effects including suppression of cccDNA were increasingly reported and thoroughly summarized elsewhere [30, 31]. Reduction of intrahepatic cccDNA was validated in vivo, consistent with the cell culture results [32]. Meanwhile, several challenges remain [33], primarily represented by off-target effects and delivery. Indeed, off-target insertions and deletions (indels) were associated with CRISPR/Cas9-mediated HBV genome inactivation [34]. Then, these editing systems have adverse events, both in terms of non-specific cleavage of host genome and integrated HBV genome [35]. In recent years, Cas9 variants, engineered Cas9 and modified guide RNAs were demonstrated to improve the specificity of the CRISPR/Cas9 system [36], and hitherto Cas9 nickases with less off-target effects were successfully harnessed against HBV [37–39]. Delivery also poses challenges, and various methods have been examined encompassing viruses, cation lipids, nanoparticles and nanomolecular DNA traps to address this limitation [40]. Notably the smaller version of Cas9 protein from Staphylococcus aureus, SaCas9, seems adapted for delivery by adeno-associated virus (AAV)-mediated delivery [41*], leading to cccDNA inactivation in vivo [42, 43]. Alternatively, small molecules that directly act on cccDNA are currently investigated. In this respect, an HBV cccDNA destabilizer (termed ccc_R08) resulted in reduced cccDNA levels in HBV-infected hepatocytes and in the liver of treated mice [44]. The nature of the molecule and its mode of action have not been disclosed yet. A second small molecule, CCC-0975, was able to reduce cccDNA biosynthesis [45]. While conceptually highly innovative, the approaches described above are largely still at the discovery or preclinical stage of development.

## Targeting Host Factors Required for cccDNA Formation and Biology

Instead of directly targeting the cccDNA, another approach is to target the host dependency factors for cccDNA formation or maintenance. Notably, cellular factors involved in the DNA repair machinery are expected to play a key role in the rcDNA-to-cccDNA conversion, and the recent identification of such key factors involved in cccDNA formation may provide promising opportunities for the development of new antivirals for HBV cure [5, 46, 47] (Fig. 1). First, an elegant study identified tyrosyl-DNA-phosphodiesterase 2 (TDP2) as a crucial factor for polymerase release from the HBV rcDNA [48*]. Through the use of multiple polymerase screening, Qi and collaborators identified the DNA polymerase K (POLK), a gamma-family DNA polymerase, as a key factor in the completion of the positive strand during DP-rcDNA conversion into cccDNA [49]. In addition, the cellular pre-mRNA processing factor 31 (PRPF31) was shown to be involved in cccDNA formation or maintenance [50]. PRPF31 interacted with HBx in the nucleus which enhanced cccDNA formation. Flap endonuclease 1 protein was shown to bind and cleave the 5’-flap structure of HBV rcDNA in vitro in order to promote cccDNA formation [51]. Furthermore, DNA ligases have been shown to be crucial for cccDNA formation [52]. By using multiple specific inhibitors and siRNA, the cellular DNA damage repair ATR-CHK1 pathway was shown to be involved in rcDNA processing and HBV cccDNA formation. Once activated, the ATR-CHK1 pathway can recruit many host DNA repair factors which are likely play a key role in cccDNA formation from rcDNA [53]. Finally, Wei and Ploss identified five core components of lagging-strand synthesis as essential to cccDNA formation: proliferating cell nuclear antigen, the replication factor C complex, DNA polymerase δ, flap endonuclease 1 and DNA ligase 1. In this study, the authors suggested that these components represent the minimal set of factors required for cccDNA formation [54*].

The redundancy of the DNA repair factors however suggests the involvement of other host factors involved in cccDNA formation and regulation [55]. Their identification is a step toward the molecular understanding of HBV persistence and toward identification of new antiviral targets. Hosttargeting antivirals have emerged as a promising approach for the treatment of viral infections, and some are clinically available, such as the chemokine receptor type 5 antagonist maraviroc for human immunodeficiency virus (HIV) treatment [56]. However, since HBV host dependency factors are also involved in the gene regulation of the host, adverse effects need to be carefully assessed [46]. These approaches are currently at the discovery stage of development.

## Silencing of cccDNA Transcription

Another therapeutic approach is the disruption of cccDNA function with silencing of viral gene transcription through the modulation of epigenetic modifications influencing cccDNA formation and control of its transcription [28, 57] (Fig. 1). Targeting epigenetic modulators is a promising approach that has already been developed and approved for cancer treatment and has been investigated against HIV and Epstein-Barr virus (EBV) infections [58, 59].

The cccDNA is organized as a minichromosome with histone and non-histone proteins and bears binding sites for various transcription factors [21, 60]. These factors include the hepatocyte nuclear factor (HNF1, HNF3, HNF4), the retinoid X receptor (RXR) and the CCAAT-enhancer-binding protein (C/EBP). Viral proteins HBx and HBc are also involved in cccDNA activity [61]. Indeed, the regulatory protein HBx is necessary for HBV transcription from cccDNA [62]. The Smc5/6 complex has been identified as an HBx interacting partner and HBV restriction factor playing a functional role in gene expression [63]. Furthermore, interferon-α (IFN-α) has been shown to reduce cccDNA-mediated transcription of viral RNA and decrease cccDNA-bound histone acetylation such as H3K9 and H3K26 marks [64, 65]. Interleukin-6 (IL-6) represses HBV replication by decreasing cccDNA-bound histone acetylation and HNF4 expression [66, 67]. The HBV genome contains three predicted CpG islands. DNA methylation on CpG islands is catalysed by DNA methyltransferases (DNMTs) in mammalian cells and generally associated with transcription silencing [68]. The involvement of DNMTs in the HBV life cycle has not been clearly elucidated yet, although methylation of CpG islands into HBV genome leads to a decreased pgRNA expression and HBV replication [69]. Targeting DNMTs could consequently constitute a strategy to inhibit cccDNA activity.

On the other hand, histone modifications such as acetylation and methylation of cccDNA-bound H3 and H4 affect cccDNA-mediated transcription. Notably, hyperacetylation and hypoacetylation of cccDNA-bound H3 and H4 histones lead to an increased and decreased HBV replication in HBV-infected patients, respectively [70*]. Furthermore, histone deacetylase 11 (HDAC11) modulates the transcription activity of cccDNA without affecting its formation by specifically decreasing the acetylation level of histone H3 [71]. Moreover, the histone deacetylase SIRT2 inhibitor AGK2 blocks cccDNA transcription in vitro and in vivo providing another therapeutic target [72]. Another study has shown that the inhibition of the HDAC KDM5 increases the H3K4Me3 and inhibits the HBV replication [73]. Finally, non-coding RNA such as microRNA (miRNA) can target and influence HBV replication by binding to HBV mRNA or by targeting host factors. For instance, microRNA-1 (miR-1) was shown to increase HBV transcription by targeting HDAC4 and E2F transcription factor 5 [74]. Long non-coding RNA (lncRNA) PCNAP1 promotes HBV replication and cccDNA accumulation by modulating miR-154/PCNA/HBV cccDNA pathway [75].

Collectively, these findings demonstrate that gene regulation of cccDNA represents an alternative therapeutic option to target cccDNA for HBV cure. However, the limitations of these strategies are the potential adverse of the different molecules and their inability to efficiently eliminate the cccDNA pool in human hepatocytes [76], thus requiring most likely long-term treatment.

## Nucleocapsid Assembly Modulators

A well-studied target of intervention is the HBV core protein (HBc) that regulates many processes in the viral life cycle such as capsid assembly, reverse transcription and virion secretion [77]. Therefore, capsid assembly modulators (CAMs) [4] (Fig. 1) have been developed to disrupt the functional roles of HBc, thereby deterring cccDNA formation. CAMs affect cccDNA levels by several mechanisms: (1) prevent reimport of newly synthetized nucleocapsid and thereby prevent amplification of the cccDNA pool [78, 79], (2) prevent formation of cccDNA in newly infected cells (probably by preventing nuclear capsid import) [80] and (3) possibly play a role in the cccDNA structure itself (since HBc is associated to cccDNA). For instance, HBc associated with CpG island 2 of cccDNA increased serum HBV DNA levels in CHB patients [81] while potentially recruiting APOBEC3A to cccDNA for its degradation [82].

Various CAMs have been developed and categorized into two major types. The first one, represented by heteroaryldihydropyrimidines (HAPs), misdirects capsid assembly (CAM-A where A stands for aberrant since these CAMs induce empty capsids with an aberrant structures), and the second one represented by phenylpropenamides (PPAs) and sulfamoylbenzamides (SBAs) induces the assembly of empty capsids (CAM-N where N stands for normal since these CAMs induce empty capsids with a normal appearance) [83]. HAPs [84–86], PPAs [87–89] as well as SBAs [90] demonstrated antiviral activities and sustained suppression of HBV DNA levels in vivo. Moreover, formation of cccDNA was shown to be inhibited by JNJ-6379 in vitro [91]. Representative HAPs (Bay 41–4109 and GLS4) and a SBA ENAN-34017 [80] also inhibited de novo synthesis of cccDNA. In recent years, discovery of new CAMs has markedly accelerated [83, 92–94]. Indeed, CAMs have been shown to disrupt amplification and formation of cccDNA [95, 96]. Several CAMs are in clinical development including JNJ-56136379 [97], JNJ-6379 [98], ABI-H0731 [99] and NVR 3–778 [100]. Long-term studies are under way to assess viral resistance and long-term virological response including cccDNA elimination.

## Immune-Mediated Degradation of cccDNA

Another appealing approach is the immune-mediated degradation of the cccDNA [28]. An interplay of innate and adaptive immunity responses is essential for viral clearance comprising both non-cytolytic and cytolytic clearance [12**]. The concept of harnessing the patient’s immune system is supported by the fact that interferon-based therapies can result in a sustained virologic response with HBsAg loss and the elimination of the cccDNA [9]. However, IFN-based therapies are limited by significant side effects, and therefore, only few patients are successfully treated. Thus, complementary approaches exploiting antiviral immune-mediated pathways are being developed [101].

In HBV-infected chimpanzees, viral clearance with reduction of cccDNA is observed in a non-cytolytic fashion [102]. Cytokines secreted by immune cells have been suggested to control non-cytolytic viral clearance [102, 103]. Intracellular pattern recognition receptors (PRRs) like toll-like receptors (TLRs) initiate the immune responses by inducting the production of antiviral cytokines and mediators such as IFNs and by inducing the activation of natural killer and T cells [104, 105]. In mouse models, HBV replication can be reduced by TLR activation. TLR3, 7/8 and 9 recognize endosomal viral nucleic acids and induce a type 1 IFN response [106]. While the TLR7 agonist GS-9620 has shown antiviral efficacy in cell culture models [107], a reduction in viremia and HBsAg in chimpanzees [108] and decrease in cccDNA levels in the woodchuck model [109], it did not result in robust antiviral effects in humans [104, 110]. Additional TLR7 agonists such as RO7020531 and JNJ-4964 are currently evaluated for CHB in clinical trials [111]. The TLR8 agonist GS-9688 substantially reduced viral DNA, RNA, antigen and cccDNA levels in the woodchuck model [111] and is now in clinical development [111]. A recent study identified IL-2 as a potential immunotherapeutic strategy able to rescue CD8+ T cells rendered dysfunctional by hepatocellular tumour initiation [112]. In addition, TRL2 was downregulated in hepatocytes, Kupffer cells and peripheral blood mononuclear cells from CHB patients [113] with an impairment of cytokine production [114]. Conversely, an increase in TLR2-positive monocytes was associated with a better response to PEG-IFN-α treatment [115*] and enhanced TLR2 expression on monocytes led to IL-6 production [116*]. In cell culture, a TLR2 ligand showed a strong anti-HBV activity [117]. Pam3CSK4 and TLR3 ligands are also efficient and may decease level of cccDNA [118]. Otherwise, degradation of cccDNA was found to be induced by apolipoprotein B mRNA editing enzyme catalytic polypeptide (APOBEC) 3 proteins. IFN-α, IFN-g, TNF-a as well as agonization of the lymphotoxin-β receptor upregulated APOBEC3A and APOBEC3B, deaminating cccDNA for its degradation [82, 102, 103]. Interestingly, overexpression of the cGAS-STING pathway reduced viral cccDNA levels in cell culture models [119]. A RIG-I agonist (inarigivir also termed SB 9200) elicited reduction in viral DNA, RNA, antigens and cccDNA in woodchucks [120] and decreased viral DNA and RNA in CHB patients [111]. However, the overall antiviral efficacy of these approaches has been limited most likely precluding their application as monotherapy.

Broad, sustained and robust antiviral T cell responses are well known to play a key role in viral cure with elimination of cccDNA in spontaneous self-limited HBV infection [121]. Thus, several strategies to improve impaired HBV-specific T cell responses in chronic HBV patients are being explored [122, 123]. Examples include therapeutic vaccines [124] and check point inhibitors (anti-programmed cell death-1 (PD-1), anti-CTLA-4) [125]. Therapeutic vaccines aim to induce or improve impaired or absent antiviral T cell responses in patient with CHB [124]. Approaches using the HBsAg-based prophylactic or vaccines with multiple viral antigens showed limited efficacy in humans [126**]. Therapeutic vaccines based on additional antigens and vectors such as modified vaccinia viruses or adenovirus have been developed and undergo clinical evaluation including prime-boost strategies [124, 127, 128]. Moreover, clinical trials for HBV viral vector vaccines and adjuvant protein vaccine (GSK3528869A) have been started (NCT03866187). While conceptually appealing, the clinical efficacy of therapeutic vaccination remains to be determined.

Immune check point proteins such as PD-1 are targeted for restoration of anti-HBV immune response breaking immune tolerance. Preclinically, the combination of an antibody to the PD-1 ligand (programmed death ligand 1) [129] with DNA vaccination resulted in complete viral clearance in a woodchuck model [130], successfully supporting the therapeutic potential of restored T cell response [131]. Indeed, blockade of PD-1 has been shown to partially restore HBV-specific T cell function [126**]. Clinical trials, e. g. for anti-PD-1 mAb nivolumab for HBV, are underway [126**]. A key challenge is an overstimulation of the immune system with severe adverse effects such as autoimmunity [125]. Moreover, reactivation of HBV infection has been observed under check point therapy [132]. While clinical studies in HBV-infected patients so far exhibited general tolerability, a risk of potentially very severe or lethal adverse effects is a clear limitation [133, 134]. Further studies are needed to understand the role of check point inhibitors in the management of CHB.

## Conclusions

Most of the current approaches aim for functional cure characterized by HBsAg loss. The ultimate goal of sterilizing cure with loss of cccDNA is desirable but much more difficult to achieve. In that regard, substantial progress has been made in the understanding of the cccDNA biology such as the identification of novel host dependency factors and previously unknown mechanisms of epigenetic regulation of cccDNA transcription. Several strategies directly or indirectly targeting cccDNA are in preclinical or early clinical development (examples shown in Table 1). Given the complexity of the HBV life cycle, it is likely that combination therapies, e. g. a combination of direct-acting antiviral(s) and immune-targeting approach(es), will be required for HBV cure including the elimination of HBV cccDNA. Further studies are needed to understand and assess the efficacy and safety of the therapeutic strategies in clinical trials. The finding that patients can spontaneously eliminate HBV infection suggests that the development of curative therapies is an achievable goal. A more detailed understanding of these mechanisms in patients may provide additional opportunities for curative therapies including elimination of cccDNA.

## Figures and Tables

**Table 1:** Examples for therapeutic strategies for HBV cure in preclinical and clinical development

Target	Concept	Stage of development	Possible challenges
Genome-editing of cccDNA	Direct cccDNA elimination	Discovery/preclinical	Off-target effects and delivery
cccDNA host dependency factors	Inhibition of cccDNA formation and/or maintenance	Discovery/preclinical	Adverse effects
Chromatin modifiers, transcription factors	Silencing of cccDNA transcription	Preclinical/clinical	Specificity for cccDNA, adverse effects, possible long-term treatment
Capsid assembly			
CAM*	HBc disruption preventing cccDNA amplification, formation and maybe playing a role in cccDNA structure itself	Clinical	Potential resistance Long-term responses unknown
Immune-mediated			
Pegylated interferon-alpha	Modulation of antiviral immune responses, cccDNA degradation	Approved	Limited response, adverse effects
PRR† agonists	Augmentation of innate responses	Clinical	Limited efficacy in monotherapy
Check point inhibitors	Restoration of T cell function	Clinical	Severe adverse effects possible
Therapeutic vaccines	Induction of antiviral T and B cell responses	Clinical	Limited efficacy in monotherapy

* *CAM* capsid assembly modulator

† *PRR* pattern recognition receptor
